# Vitamin D3-Coated Surfaces and Their Role in Bone Repair and Peri-Implant Biomechanics

**DOI:** 10.3390/biology14050476

**Published:** 2025-04-26

**Authors:** Letícia Pitol-Palin, Isadora Castaldi Sousa, Juliani Caroline Ribeiro de Araújo, Fábio Roberto de Souza Batista, Bruna Kaori Namba Inoue, Paulo Roberto Botacin, Luana Marotta Reis de Vasconcellos, Paulo Noronha Lisboa-Filho, Roberta Okamoto

**Affiliations:** 1 Department of Diagnosis and Surgery, Araçatuba Dental School, São Paulo State University, Araçatuba 16015-050, Brazil; kaori.namba@unesp.br; 2 Department of Basic Sciences, Araçatuba Dental School, São Paulo State University, Araçatuba 16066-840, Brazil; isadora.c.sousa@unesp.br (I.C.S.); fabiorsbatista@gmail.com (F.R.d.S.B.); paulo.botacin@unesp.br (P.R.B.); 3 Department of Biosciences and Oral Diagnosis, Institute of Science and Technology, São Paulo State University, São José dos Campos 12245-000, Brazil; juliani.ribeiro@unesp.br (J.C.R.d.A.); luana.marotta@unesp.br (L.M.R.d.V.); 4 Department of Physics and Meteorology, Bauru Sciences School, São Paulo State University, Bauru 17033-360, Brazil; paulo.lisboa@unesp.br

**Keywords:** vitamin D3, bone remodeling, bone matrix, dental implants, animal model

## Abstract

Dental rehabilitation with titanium implants may requires the optimization of techniques and materials when oral conditions affect the successful treatment result. Thus, this study aims to customize the surface of titanium implants with bioactive vitamin D3 molecules to increase the performance of bone repair. The surfaces were functionalized following the “dip-coating” incorporation method with vitamin D3 in a solution of 1000 I.U./goat. The work was carried out in two stages: (I) physicochemical and biological tests (in vivo) in order to characterize and validate the vitamin D3 surface as well as its ability to affect peri-implant bone biomechanics; and (II) in vitro experiments to characterize viability responses, interaction and cell mineralization capacity. Scanning electron microscopy showed that the creation of vitamin D3 films is stable and homogeneous, while the in vivo results showed an increase in the biomechanical and microarchitectural capacity of the bone when vitamin D3 implants were used. Furthermore, the application of functionalized surfaces proved effective in promoting cell interaction and bone mineralization processes while preserving cell viability and capacity. In conclusion, the delivery of bioactive molecules based on vitamin D3 promotes changes in the surface microstructure of titanium, enabling an increase in the structural characteristics of bone tissue that result in an improvement in bone repair and peri-implant biomechanics.

## Simple Summary

This study explores the use of nanotechnologies applied to the surfaces of dental implants to optimize the bone repair process in normo-physiological rats. Thus, an administration system based on drug delivery was developed using vitamin D3 biomolecules in two concentrations (vD40µl and vD400µl) for physicochemical and in vitro characterization and, in addition, to observe their bone responses in an animal model. The physicochemical results showed the appropriate physical conditions obtained by creating a stable film with a uniform presence of biomolecules. In addition, animal tests showed that the use of vitamin D3-functionalized surfaces promoted an increase in biomechanical capacity and peri-implant bone repaired microarchitecture.

## 1. Introduction

Nanotechnology applied to the implant surface is a viable strategy to optimize the biomechanical properties of bone during the reparational process [1,2,3,4,5,6,7]. Rehabilitation of patients with systemic complications or even severe bone loss requires the optimization of techniques and materials, where geometric modifications are not able to imply a successful treatment result. Thus, modifying the morphology of titanium (Ti) and Ti-based surfaces, especially by physicochemical methods with biomolecules, has been a subject of considerable relevance in the dental community [1,2,3,4,5,6,7].

In this context, the drug delivery (DD) system is an alternative method of supplying bioactive molecules. This controlled liberation system consists of manufacturing a device able to release a continuous rate of the compound over an extended duration [8]. Thus, it is possible to create a predictable liberation pattern based on (I) biocompatibility, (II) directional biodistribution, (III) stability and half-life and (IV) functionality [9]. The main advantages of the DD-based delivery system include a reduction in the adverse effects of the medication, since it is administered locally, and the possibility of personalizing the treatment program by selecting the biomolecule according to the objective of the specialist [8,9,10,11,12].

Vitamin D3 (vD; cholecalciferol) is a steroidal hormone and plays an important role in calcium and phosphate metabolism, acting directly on bone turnover through the activation of osteoclasts and osteoblasts [13,14,15]. In addition, vD has a fundamental role in the regulation of immune system cells (dendritic cells, macrophages and T and B cells), increasing expression of anti-inflammatory proteins and decreasing pro-inflammatory ones, contributing to bone turnover [16,17,18]. In this context, adequate levels of vitamin D can improve tissue repair by enhancing the bone microarchitecture around Ti implants, promoting a more efficient and accelerated osseointegration.

Thus, the main hypothesis of this study concerns the effects of vitamin D3 biomolecules applied to titanium in a drug delivery system, which could control the local inflammatory response inherent in surgical procedures during implant installation, accelerating the osseointegration process. Therefore, this study aims to customize the surface of titanium implants with bioactive vitamin D3 molecules to increase the performance of bone repair.

## 2. Materials and Methods

The work was carried out in two stages: (I) physicochemical and biological tests (in vivo) in order to characterize and validate the vitamin D3 surface as well as its ability to affect peri-implant bone biomechanics; and (II) in vitro experiments to characterize viability responses, interaction and cell mineralization capacity.

### 2.1. Titanium Implants and Discs

Titanium grade IV implants with a double acid etching were personalized (2 mm diameter × 4 mm height; TitaniumFix^®^, São José dos Campos, SP, Brazil) and sterilized by gamma rays. The titanium discs also had the same surface characteristics (8 mm diameter × 2 mm thickness).

### 2.2. Vitamin D3 Surface—Coating Technique

The surfaces were functionalized with vitamin D3 in a solution of 1000 I.U./goat (ADDERA D3^®^, Mantecorp Farmasa. São Paulo, São Paulo, Brazil) diluted in dimethyl sulfoxide (DMSO—Labsynth^®^ Produtos Químicos, Matérias-Primas, Reagentes Analíticos, Equipamentos e Acessórios de Laboratórios. Diadema, São Paulo, Brazil). In order to define the ideal dose, two concentrations of vitamin D3 were selected for physicochemical tests: a vD40 surface (40 µL of vitamin D3 in 100 mL of DMSO—1 drop of Addera D3^®^) and vD400 surface (400 µL of vitamin D3 in 100 mL of DMSO—10 drops of Addera D3^®^). To determine the concentrations, a bibliographic review was carried out, selecting the most frequently used concentration (transformed into a solution in drops) and, for comparison, a 10× more concentrated solution.

The surfaces were functionalized following the “dip-coating” incorporation technique, which consists of immersing the implants in a solution in order to create bonded coats through physical—chemical connections among the layers. To create the surface coating on the implants and discs, five immersions in vitamin D3 solution were carried out, as mentioned above. The implants were initially attached to micropipette tips to facilitate handling without directly manipulating the treated surfaces. A 15-min exposure to UVC light was carried out before immersion in the vitamin D3 solutions. The implants and discs were introduced perpendicular to the solution to reduce surface tension and ensure complete surface wettability. The implants and discs were kept in contact with the solution for an increasing exposure time to ensure adhesion between the layers: 1st immersion—5 s; 2nd immersion—10 s; 3rd immersion—15 s; 4th immersion—20 s and 5th immersion—25 s. Air humidity control was maintained between immersions until the surfaces were completely dry [1,19,20]. Before the implants’ installation in the animals, a 15 min exposure to UVC light was carried out.

### 2.3. Physicochemical Tests

Scanning Electron Microscopy (SEM): The microstructural characterization of the treated surfaces was carried out using a scanning electron microscope with an electrostatic emission source (FEMEV, Zeiss-SUPRA35. Zeiss, Oberkochen, Germany) operated at 5 kV at different magnifications. To take the images in scanning mode, the surfaces were previously prepared by adding a small amount of the sample to a beaker containing acetone to be subjected to ultrasound treatment for 15 min. Then, 2 drops of the solution were deposited on a Si substrate which was duly glued to the sample holder (stub) with carbon tape. After the solution had dried on the substrate, electrical contact was made by applying conductive silver paint to one end of the substrate. Transmission electron microscopy (TEM) and high-resolution electron microscopy (HRMET) analyses were carried out on a JEOL microscope (JEM, 2100 HT) operated at 200 Kv [1,19,20].

### 2.4. In Vivo Tests

Animals: The study was approved by the Ethics Committee for the Use of Animals (CEUA) of the Araçatuba Dental School (FOA/UNESP #00129-2021, approved on 24 June 2021), following the Animal Research: Reporting of In Vivo Experiments (ARRIVE) guidelines [21]. Fifteen 3-month-old male rats (Rattus norvegicus albinus, Wistar) were divided into three groups (*n* = 5 per group), with the according surfaces: titanium surface (Ti), vD40µl surface (vD40) and vD400µl surface (vD400). The animals were kept in cages, identified and randomly separated based on previous results already published [22]. The averages used for the calculation were 3.06 and 4.898 and the standard deviations were 0.26 and 0.024, with a significance level of 5% and a power of 95% in a one-tailed hypothesis test.

Implant Placement: The animals were anesthetized with xylazine hydrochloride and ketamine hydrochloride to perform the antisepsis and surgical procedure according Wajima et al. 2024 [22]. Surgical access was obtained with a 1.5 cm incision in the tibial metaphysis. The soft tissue in the tibial metaphysis was then dissected, to expose the bone surface to receive the implants. The implants were installed bilaterally in each tibia with bicortical stabilization, following the preestablished groups. The sutures were made with Vycril and, in the immediate postoperative period, each animal received pentabiotic and sodic dipyrone [22].

Peri-implant Biomechanical Analysis: The animals were euthanized by anesthetic overdosage 28 days after the implant installation [22]. The tibia was accessed to expose the implants and a digital torque meter coupled with a 0.9 mm hexagonal digital key was used. To measure the removal torque of the implants, a counterclockwise movement was performed until the implants rotated completely, and the maximum force value obtained (N.cm) was registered [22].

**Figure 1:**
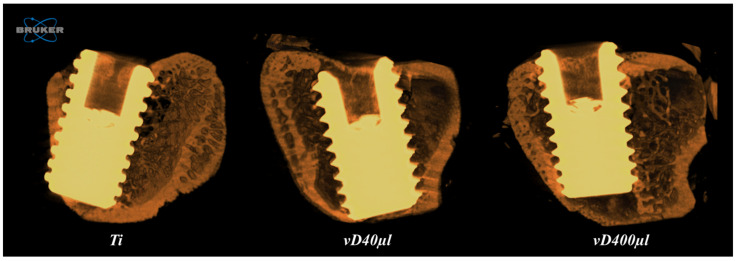
Three-dimensional reconstruction of the peri-implant region of the Ti, vD40 and vD400 groups. Images obtained by the CTVox 3.3 software through microtomographic analysis.

Microtomography Analysis (Micro-CT): The samples were scanned by a SkyScan 1272 microtomograph (SkyScan 1272 BrukerMicroCT, Kontich, Belgium), reconstituted, corrected and evaluated according to Shirazi et al. 2024 [4]. The CTAnalyser-CTAn software (2003-11SkyScan, 2012 BrukerMicroCT Version 1.12.4.0) was used to evaluate the 3D extension of the tibial metaphysis, selecting 100 slices to perform the analysis. Implant and bone regions of interest (ROI) were determined for the same volume. A binary threshold was performed with a white value of 90–255, selecting only the titanium implant, followed by removal of pores and dilatation by 10 pixels. This image was considered as the region of interest one (ROI 1) and again was dilated, but now by 20 pixels (this new threshold was named as IMAGE), and then the IMAGE was subtracted by ROI 1, and the result of this was set as the final ROI. A new threshold was loaded to evaluate and isolate the bone, this time using grayscale values of 20–255, and this final volume of interest (VOI) was evaluated by 3D analysis of the peri-implant bone marrow. Thus, we were able to define the percent bone volume (BV/TV), trabecular thickness (Tb.Th), trabecular number and separation (Tb.N, Tb.Sp), bone-to-implant contact (BIC) through the intersection surface and total porosity (Po.Tot) [23] (Figure 1).

### 2.5. Mesenchymal Cell Culture and Cell Differentiation Analysis

*Culture isolation and primary culture of osteogenic cells:* The cells were obtained from the femurs of the rats (CEUA 03/2021) and the analysis was performed according to Andrade et al. 2015 [24]. All the tests were carried out in accordance with ISO 10993-5 [25] and in triplicate, with each triplicate being a pool of cells from the femurs of 4 animals from each group. The control group used in all tests was the titanium surface with double acid etching. The short-term cell response was evaluated by cell adhesion, proliferation, viability and morphology, whereas the long-term cell response was assessed by osteoblastic differentiation using measures of alkaline phosphate (ALP) activity and quantification of matrix production, according to previous publications [24,26,27,28].

### 2.6. Statistical Analysis

The GraphPad Prism 7.03 software (GraphPad Software, La Jolla, CA, USA) was used for statistical analysis. To confirm normal distribution, the Shapiro–Wilk test was used in order to verify the homoscedasticity of the data. The one-way ANOVA test was used to determine whether there were significant differences between the groups. For direct comparisons between groups, the Tukey post-hoc test was applied. A significance level of 5% was applied to all analyses.

## 3. Results

### 3.1. Scanning Electron Microscopy (SEM)

In order to define the surface functionalization, two concentrations of Addera D3^®^ were selected for physicochemical tests and implant installation in a pilot group, to assess which of the two concentrations would have a better effect on the implant osseointegration process. A comparison was therefore made between the conventional titanium surface, the vD40 surface (40 µL of vitamin D in 100 mL of DMSO—1 drop of Addera D3^®^) and the vD400 surface (400 µL of vitamin D in 100 mL of DMSO—10 drops of Addera D3^®^). SEM was used to characterize the surfaces treated with the two proposed concentrations of Addera D3^®^, vD40µl and vD400µl, in order to observe the morphological and adhesion profiles of the vD treatment.

**Figure 2:**
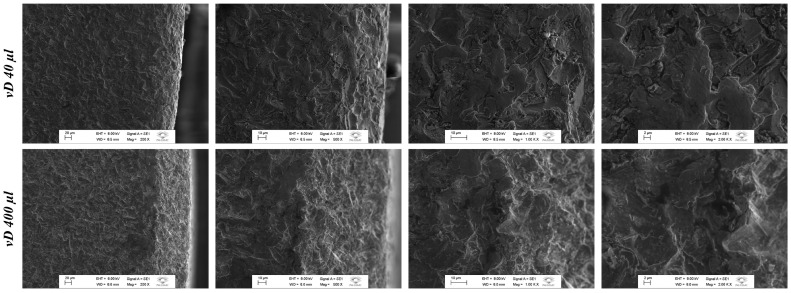
SEM of titanium discs functionalized with vitamin D3. SEM photomicrographs of the vD40 surface (40 µL of vitamin D in 100 mL of DMSO—1 drop of Addera D3^®^) and the vD400 surface (400 µL of vitamin D in 100 mL of DMSO—10 drops of Addera D3^®^). Magnification: 200×, 500×, 1k× and 2k×.

It is possible to observe the greater viscosity (adherence) and morphology of the vD400µl surface, fundamental characteristics for the stability of the layers created by the dip-coating technique (Figure 2).

### 3.2. Microtomography Analysis (Micro-CT)

**Figure 3:**
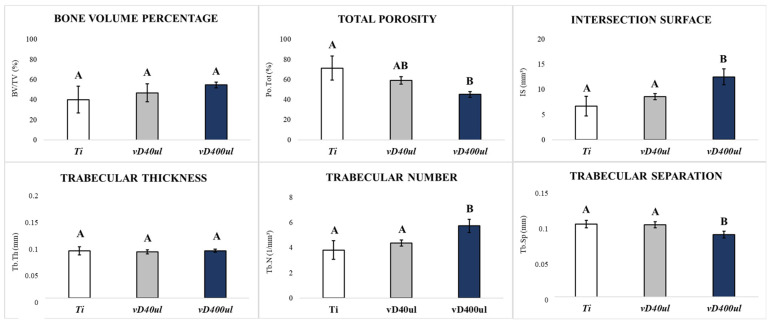
Microtomographic analysis of the comparison between the Ti, vD40µl and vD400µl surfaces. Graphs show the mean values obtained in the microtomographic analysis between the Ti, vD40µl and vD400µl surfaces at 28 days of peri-implant repair. Letters correspond to the comparison between the groups. One-way ANOVA statistical test (*p* < 0.05).

Bone Volume Percentage (BV/TV): There was no statistically significant difference when comparing the surfaces (*p* = 0.1446). However, the group with the vD400µl surface showed higher numerical values (54.7%) compared to the vD40µl (46.8%) and Ti (40.1%) groups (Figure 3; Table 1).

Total Porosity (Po.Tot): There was a statistically significant difference when comparing the vD400µl vs. Ti surfaces (*p* = 0.0020), where the lowest values of tissue porosity are presented on the vD400µl surface (45.32%), followed by the vD40µl (59.26%) and Ti (61.43%) surfaces (Figure 3; Table 1).

Intersection Surface (IS): There were statistically significant differences when comparing the vD400µl vs. Ti surface (*p* = 0.0010) and vD400µl vs. vD40µl (*p* = 0.0119), where the highest values of bone formation in contact with the implant are presented on the vD400µl surface (Figure 3; Table 1).

Trabecular Thickness (Tb.Th): There was no statistically significant difference when comparing the Ti, vD40µl and vD400µl surfaces (*p* = 0.8217) (Figure 3; Table 1).

Trabecular Number (Tb.N): There were statistically significant differences when comparing the vD400µl vs. Ti surface (*p* = 0.0018) and vD400µl vs. vD40µl (*p* = 0.0146), where the highest values of trabecular number per mm are presented on the vD400µl surface (Figure 3; Table 1).

Trabecular Separation (Tb.Sp): There were statistically significant differences when comparing the vD400µl vs. Ti surface (*p* = 0.0043) and vD400µl vs. vD40µl (*p* = 0.0071), where the lowest trabecular separation values were found for the vD400µl surface (Figure 3; Table 1).

### 3.3. Peri-Implant Biomechanical Analysis

The conventional Ti surface showed lower removal torque values, while the highest values were obtained for the vD400µl surface. In the statistical comparison, there was a significant difference between Ti vs. vD400µl (*p* = 0.0334). The comparison between Ti vs. vD40µl (*p* = 0.1091) and between vD40µl vs. vD400µl (*p* = 0.7469) showed no statistically significant difference between the results obtained (Table 2).

### 3.4. Mesenchymal Cell Culture and Cell Differentiation Analysis

**Figure 4:**
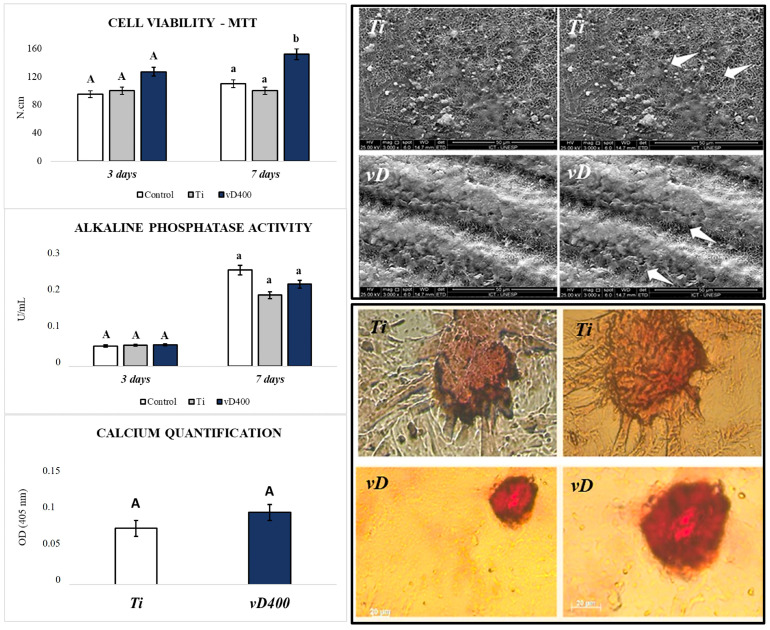
Cell culture and differentiation analysis. Results obtained in cell culture analysis, where the vD400μl surface showed an increase in viable cells interacting with the culture medium. In addition, the vD400μl surface promoted an increase in ALP activity and calcium quantification compared to the Ti surface. Letters correspond to the comparison between the groups. One-way ANOVA statistical test (*p* < 0.05).

Cell Viability Determination: At 3 and 7 days, a quantitative evaluation of live cells was carried out after exposure to the MTT stain and spectrophotometric analysis of the incorporated dye. At 3 days, there was no statistically significant difference when comparing the Ti vs. vD400µl surface (*p* > 0.05). At 7 days, there was a greater quantity of live mesenchymal cells, with a statistically significant difference, in contact with the vD400µl surface compared to the Ti surface (*p* < 0.05) (Figure 4).

Cell adhesion and proliferation: After 3 days of culture, the cell morphology was evaluated by SEM-FE to evidence cell interaction on the materials. The analysis showed that all the samples enabled cell spreading, although the materials had irregular macrostructures and a rough surface. It was also possible to observe cell extensions permeating the surface of the material (Figure 4).

Alkaline phosphatase activity: Alkaline phosphatase activity was evaluated on the Ti and vD400µl surfaces at 3 and 7 days. At 3 days, there was no statistically significant difference between the surfaces (*p* > 0.05). However, at 7 days, although there was no statistically significant difference when comparing Ti vs. vD400µl (*p* > 0.05), there was a discrete increase in ALP activity on the vD400µl surface (Figure 4).

Mineralized bone-like nodule formation: The formation of mineralization nodules was assessed after 14 days of culture using 2% Alizarin red S staining. After quantification, a greater amount of calcium was observed in the nodules formed on the vD400µl vs. Ti surface (*p* > 0.05) (Figure 4).

## 4. Discussion

The development of technologies and local therapeutic approaches that are able to optimize physiological responses, as well as the results obtained in the rehabilitation process, remains a subject of great scientific interest in dentistry. Nano-loading of drugs can be performed directly on titanium implants with immediate and constant delivery to the bone tissue. This solution can provide a controlled distribution of biomolecules in the bone microenvironment and is a simple and effective approach to different types of concerns.

Through the incorporation of biomolecules, the technique used can influence the morphology and size of the particles, as well as the roughness and wettability of the surface [29]. The choice of the dip-coating technique offers the advantages of ease and low cost, as well as allowing a good distribution of particles and thickness of the film formed, and providing an easily reproducible process [29]. Dip-coating is a form of Layer-by-Layer where molecules can bond to the surface of titanium and its thin layer of titanium oxide (TiO_2_) through a physical/mechanical bond as well as chemical bonds (covalent bonds) [29]. TiO_2_ has hydroxyl groups (-OH) in its structure and, under environmental conditions, this leads to covalent interactions between -OH and a specific functional group present in organic molecules [30], such as vitamin D3 or the DMSO substrate used in this study. There is also another terminal functional group free to interact with other molecules on the surface, with useful applications for bioengineering [29].

In this study, the creation of five layers creates an adequate thickness with stable bonds for the installation of the implants. To create the first layer, the use of UVC light hydroxylates the TiO2, providing covalent bonding sites [29] in conjunction with the mechanical adhesion of the vitamin D3-based solution. DMSO is also a favorable choice for creating layers [31,32], since it has an ideal viscosity for mechanical bonding between the layers [31,32], as well as having antimicrobial biological effects and offering biocompatibility [31]. Meanwhile, it is not possible to understand how these layers remain stable after the implants have been installed. This is due to the fact that it is not possible to perform SEM with the implants installed in the animals, since this would require the removal of the implants, impacting on increased film loss.

In vitro tests show positive characteristics for the incorporation technique with vD400µl on the titanium surface, indicating that the use of functionalized implants did not promote cell damage, aiding in the process of bone formation and mineralization. In addition to these in vitro results, the increase in calcium quantification is a factor that indicates an increase in the mineralization process. A study [33] with human mesenchymal stem cells showed that vitamin D3 is also able to modulate the differentiation responses of osteoblast lineages in the growth culture medium. These factors are essential for the development of a biocompatible material that acts in a beneficial way and interacts with the cellular environment, aiding the osseointegration process and the longevity of implants [34]. A similar situation is found using other technologies applied to the titanium surface, such as macro-geometry modifications [35], the creation of TiO_2_ nanotubes [36,37] and through the additive manufacturing technique [38,39].

The physical and biological characteristics of the vitamin D3-coated surface had positive results on the peri-implant response observed through the in vivo results. The evaluated surfaces (Ti, vD40µl and vD400µl) showed similar results in bone microarchitecture parameters; however, the use of vD400µl implants promoted a uniform clinical result among the animals, as well as increasing BV/TV, Tb.N and having a significant impact on the bone/implant contact area. Trabecular parameters are taken into account when referring to the quality of bone tissue [23], since an appropriate number and thickness of trabeculae are essential characteristics for the development of mechanically appropriate bone tissue. The mechanical comportment of trabecular bone is strongly influenced by microarchitectural characteristics, while the comportment of cortical bone is mainly dominated by material properties [40]. A study with eldecalcitol [41], one of the vitamin D3 forms, showed that its systemic administration promoted prevention of trabecular bone loss as well as bone fragility in diabetic rats, corroborating the results of the present study. This indicates that the use of vitamin D3 biomolecules was able to aid the formation of trabecular bone, the type of bone found in the maxillofacial area.

Quality factors in bone tissue have a direct impact on bone biomechanics, since deficiencies in the characteristics of bone microstructure will impair the ability of this tissue to support external forces [42]. The study of the strength of trabecular bone plays an important role, since it may be related to the injuries caused by bone remodeling and bone implant failure [42]. Trabecular bone is important as a load-bearing organism [42]. It is also related to force and affects the rupture risk of the bone structure, or the failure of titanium implants [43]. The biomechanical properties of the peri-implant bone were measured through implant removal torque, enabling an assessment of the load (N.cm) supported by reparational bone 28 days after the installation of vitamin D3-based or conventional titanium implants. Vitamin D3 coating also led to an increase in the parameter used to assess peri-implant biomechanics, observed through an increase in implant removal force, especially in vD400μl.

Vitamin D3 has already been the subject of many studies considering its systemic action [44,45,46,47,48]. In an orchiectomized rat model, treated systemically with vitamin D, the medication had a positive systemic effect on the tissue repair process [48]. However, the dose of vitamin D3 used should be considerably increased, and in many cases, it does not achieve satisfactory results in terms of peri-implant biomechanics. Drug delivery systems improve safety and efficacy using a reduced concentration of drugs [49,50,51] in the peri-implant repair context, since it is possible to enhance the biomechanical capacity of restorative bone tissue without the systemic use of pharmaceutical therapies.

The development of biotechnologies applied to biomaterials has been crucial to the advancement of reconstructive and rehabilitative treatments in dentistry. The results found in this study show the importance of the structural quality of trabecular bone, serving not only as a support for correct bone formation and mineralization, but also to withstand the mechanical forces that affect bone tissue. Thus, the presence of biomolecules applied to titanium promoted an increase in the biomechanical capacity of bone tissue, due to their direct action on the quality of the tissue matrix, as well as on the characteristics of the trabecular bone microstructure. These results suggest new perspectives for the use of biomolecules to accelerate the bone repair process in systemically healthy patients, as well as for the local treatment of systemically compromised patients and for those with severe tissue damage.

## 5. Conclusions

Drug delivery of bioactive molecules based on vitamin D3 promotes changes in the surface microstructure of titanium, without impacting on cell viability and function, enabling an increase in the structural characteristics of bone which results in an improvement in bone repair and peri-implant biomechanics.

## Figures and Tables

**Table 1:** Average and standard deviation of the values obtained from the parameters evaluated in the microtomographic analysis.

	Ti	vD40µl	vD400µl
BV/TV (%)	40.1 ± 13.28	46.8 ± 8.97	54.7 ± 2.87
Po.Tot (%) ^†^	61.43 ± 12.01	59.26 ± 3.61	45.32 ± 2.87
IS (mm^2^) ^†^	6.71 ± 1.95	8.62 ± 0.61	12.54 ± 1.56
Tb.Th (mm)	0.094 ± 0.008	0.092 ± 0.004	0.094 ± 0.003
Tb.N (1/mm^2^) ^†^	3.82 ± 0.73	4.38 ± 0.23	5.75 ± 0.52
Tb.Sp (mm) ^†^	0.108 ± 0.005	0.107 ± 0.004	0.092 ± 0.005

One-way ANOVA statistical test. ^†^: *p* < 0.05.

**Table 2:** Average and standard deviation of the values obtained from peri-implant biomechanical analysis.

Ti	vD40µl	vD400µl
6.98 ± 1.181 ^A^	8.91 ± 2.091 ^AB^	9.65 ± 1.526 ^B^

One-way ANOVA statistical test. Letters correspond to the comparison between the groups. One-way ANOVA statistical test (*p* < 0.05).

## Data Availability

The data used are contained within the article.

## Abbreviations

The following abbreviations are used in this manuscript:

TLA	Three letter acronym
LD	Linear dichroism
Ti	Titanium and titanium surface
vD	Vitamin D3
vD40µl	Vitamin D40µl surface
vD400µl	Vitamin D400µl surface
SEM	Scanning electron microscopy
DD	Drug delivery systems
MTT	3-(4,5-dimethylthiazol-2-yl)2,5-diphenyltetrazolium bromide
ALP	Alkaline phosphatase
